# Knowledge and attitude factors associated with the prevalence of Tdap (tetanus, diphtheria, and acellular pertussis) booster vaccination in healthcare workers in a large academic hospital in Southern Italy in 2022: a cross-sectional study

**DOI:** 10.3389/fpubh.2023.1173482

**Published:** 2023-07-13

**Authors:** Michelangelo Mercogliano, Claudio Fiorilla, Federica Esposito, Michele Sorrentino, Pasquale Domenico Mirizzi, Antonio Parisi, Andrea Tajani, Gaetano Buonocore, Maria Triassi, Raffaele Palladino

**Affiliations:** ^1^Department of Public Health, University “Federico II” of Naples, Naples, Italy; ^2^Clinical Directorate, University Hospital “Federico II” of Naples, Naples, Italy; ^3^Interdepartmental Research Center in Healthcare Management and Innovation in Healthcare (CIRMIS), Naples, Italy; ^4^Department of Primary Care and Public Health, School of Public Health, Imperial College, London, United Kingdom

**Keywords:** Tdap, vaccine, pertussis, knowledge, questionnaire, attitude, booster, healthcare

## Abstract

**Introduction:**

In Europe, there is still suboptimal tetanus, diphtheria, and acellular pertussis (Tdap) booster coverage. This study aimed to assess coverage status, knowledge, and attitude on Tdap vaccination in healthcare workers (HcWs) of the University Hospital “Federico II” in Naples, Southern Italy, in 2022, to improve current vaccination strategies.

**Methods:**

A cross-sectional study was conducted using a validated anonymous questionnaire. Knowledge and attitude were measured as scores. Multivariable logistic and linear regression models were employed to identify correlates of Tdap booster and knowledge and attitude toward the vaccination, as appropriate. Models were controlled for age, sex, profession, department, and job seniority.

**Results:**

A total of 206 questionnaires were administered among HcWs, and 143 (69.4%) were medical doctors. In total, 71 (34.47%) HcWs received the Tdap booster. Those who have worked 5–9 years at the hospital had a 78% lower likelihood of being vaccinated with the Tdap booster (5–9 years—OR: 0.22, CI: 0.06 | 0.85) as compared with newly hired HcWs. No differences in the average knowledge score were found. Other healthcare workers had a lower attitude as compared to medical doctors (Other—Coef. −2.15; CI: −4.14 | −0.15) and, as compared with those who worked in a clinical department, those who worked in a diagnostic–therapeutic department or medical management had 3.1 and 2.0 lower attitude scores, on average, respectively (diagnostic–therapeutic—Coef. −3.12, CI: −5.13 | −1.12; public health—Coef. −1.98, CI: −3.41 | −0.56).

**Discussion:**

The study findings support the necessity to implement public health strategies and improve knowledge and attitude toward vaccinations and specifically highlight the importance of Tdap booster every 10 years as a prevention tool to protect high-risk populations.

## Introduction

The burden of vaccine-preventable diseases is still a global concern. In the decade 2010–2019, epidemic outbreaks of pertussis have been reported in several countries worldwide (1), although this figure is in contrast with what has been observed in the past 3 years. For instance, in 2021, pertussis cases almost halved compared with previous years (2). In Europe, the cases reported in 2021 were 2,157 compared with more than 12,000 in 2020 (3). However, the main factor responsible for the observed reduced incidence in this period is likely to be the implementation of non-pharmaceutical interventions (NPI) to reduce the impact of the COVID-19 pandemic on Health Systems (e.g., the use of filtered masks, continuous hand hygiene, and contact ban) rather than specific preventive strategies for pertussis, such as tetanus, diphtheria, and acellular pertussis (Tdap) vaccination (4, 5).

Data provided by the World Health Organization show that globally the coverage of vaccination against pertussis among 1-year-old children has decreased from 2019 to 2021 by 5% (from 86 to 81%), with an estimated loss of approximately 25 million pediatric vaccinations (6). In Europe, although the reduction was more contained, a drop between 1 and 5% in the 0–24 months and 0–6 years of vaccination coverage between 2018 and 2021 has been documented (7). This reduction in vaccination coverage is worrisome and might considerably impact population health in the upcoming years of transition from pandemic to endemic. Despite a strong initial reduction in the incidence of respiratory infectious diseases, the implementation of NPIs has only a transient effect, with a backlash effect when lifted (8).

In Italy, according to the national vaccination plan, the primary cycle and the booster doses are provided free of charge (9), the official 2021 National Health System data reported that the average coverage for Tdap vaccinations in the 0–24 months population was 94 and 72–73% for the 0–18 years booster coverage, below the WHO threshold and with profound inter-regional differences (10).

The perdurance of vaccine protection is not established, hence, booster dose coverage is pivotal. Numerous studies evidence decreasing levels of anti-pertussis immunoglobulin G over time from vaccination, suggesting that immunity wanes in the years following the last dose of Tdap (11).

In Italy, tetanus, diphtheria, and acellular pertussis booster doses are recommended for adolescents and then every 10 years in adults to reduce the transmission and to protect the community, especially since Italy, in 2018, accounted for 39.1% of all notified cases of Tetanus in EU/EEA countries (11–14). Furthermore, in Italy, cases of pertussis have increased from 503 to 962 during 2015–18 (15), with a strong likelihood to be underreported (16). The implementation of the active offer to professional categories at risk is particularly important, given the high contagiousness of infectious diseases, such as pertussis to newborns, who have not yet been vaccinated (17).

Although healthcare workers (HcWs) are a target group to achieve high vaccination coverage (18), they usually show a low awareness of work-related risks (19) and can be a source of infection for susceptible patients and relatives, as well as other HcWs (20–23).

Despite the importance of reaching immunization targets for HcWs, there is a paucity of evidence related to the topic. A systematic review conducted in 2019 found only 28 studies that examined Tdap coverage on HcWs; in the included studies, the highest coverage rate observed was 63.9%, despite that, on average was just 40.0% (24).

This study aimed to estimate the Tdap coverage status in HcWs at the University Hospital “Federico II” in Naples, a large university hospital in Southern Italy, in 2022 and to assess knowledge and attitude on Tdap vaccination and their correlates to improve current vaccination strategies and implement prevention counseling in health surveillance.

## Methods

### Study design

This cross-sectional study has been conducted to estimate Tdap coverage, knowledge, and attitude toward vaccinations in HcWs. Data were collected through the administration of an anonymous questionnaire. All HcWs at the University Hospital “Federico II” of Naples, the largest university hospital in Southern Italy, were invited to participate in the study between October and December 2022. The study was approved by the University Hospital Ethical Committee (Prot. N. 00018993–11/08/2022) and conducted in accordance with good clinical practice and the Declaration of Helsinki.

### Study variables

Study variables were retrieved from a questionnaire that was adapted from a previously validated questionnaire (25). Before the questionnaire administration to our target population, it was discussed by a focus group composed of physicians and other healthcare workers to evaluate its comprehensibility and intelligibility. The questionnaire in its final form is available in the Supplementary Figure 1.

Study variables included the following sociodemographic characteristics: sex (male and female), age (up to 34 years old, 35 years, and older), and educational attainment (high school and below and degree and above). Additional variables related to the job status were as follows: profession (medical doctors, non-medical healthcare workers, such as nurses and healthcare assistants, and other healthcare workers including biologists and administrative staff), department (clinical, surgery, diagnostic–therapeutic, and medical management), job seniority (0–4 years, 5–9 years, and more than 10 years), and vaccination history (vaccinated against measles, mumps, rubella, hepatitis B, polio, chicken pox, *Haemophilus influenzae*, and tuberculosis; coded as yes/no/not sure). For vaccination history, a score of 3 was assigned to the answer “yes”, 2 to “not sure”, and 1 to “no” (26). Based on these answers, we constructed a score ranging from 8 to 24.

Outcome variables included the Tdap booster coverage in the past 10 years and the attitude and knowledge about vaccines. The knowledge section included 15 questions regarding recommended vaccinations. A score of 3 was assigned to the answer “yes”, 2 to “not sure”, and 1 to “no” (26). Based on these answers, we constructed a score ranging from 15 to 45. Attitude toward recommended vaccinations was measured as a score (ranging from 3 to 30) obtained through three questions regarding the perception of the risk of contracting an infection and the usefulness of vaccination for HcWs to protect themselves and patients. Each question comprised a scale from 1 to 10. The final score was obtained by summing up the three values.

### Statistical analyses

Study population characteristics were summarized using descriptive statistics, as appropriate. Multivariable regression models controlled for gender, age, profession, education, department, and job seniority were employed to assess correlates of vaccination coverage, knowledge, and attitude. To better assess the contribution of each variable, we first controlled the regression model for gender and age (partially adjusted model), then we also included education, job, department, and job seniority (fully adjusted model). Only for boosters, we also considered a third model including knowledge, attitude, and vaccination history. Specifically, multivariable logistic regression models were employed for binary outcomes and linear regression models for continuous outcomes. The results are presented as odds ratios (ORs), statistical coefficients (Coef.), and 95% confidence intervals (95%CIs), as appropriate. The results were considered significant if the *p*-value was <0.05. Statistical analyses were performed using Stata MP 15.0 statistical software.

## Results

During the study period, 206 questionnaires were completed. The demographic characteristics of participants are presented in Table 1. In total, 50% of the sample participants were women: 26.8% were <35 years old and 43.2% were ≥35 years old. The majority of the sample participants, 93.7% (193), had a degree or higher education and 6.3% (13) did not. As per the job status, 69.4% of the subjects (143) were medical doctors, 18.9% (39) were non-medical HcWs, and the remaining workers were 11.6% (24). In total, 32.0% of the study population (66) was working in a clinical department, 15.0% (31) in a surgical department, 10.2% (21) in a diagnostic–therapeutic department, and the remaining 42.7% (88) in a medical management department. Most of the subjects, 74.8% (154), had worked for the university hospital for 0–4 years, 12.1% (25) for 5–9 years, and the remaining 13.1% (27) for 10 or more years.

**Table 1:** Characteristics of the study population.

**Study population**	***N***	**Percentage**
Sample size	206	
**Sex**		
Male	103	50.00
Female	103	50.00
**Age**		
<35 years	117	26.80
≥35 years	89	43.20
**Education**		
Less than degree	13	6.31
Degree or higher	193	93.69
**Profession**		
Medical doctors	143	69.42
Non-medical healthcare workers	39	18.93
Others healthcare workers	24	11.65
**Department**		
Clinical	66	32.04
Surgery	31	15.05
Diagnostic–therapeutic	21	10.19
Medical management	88	42.72
**Job seniority (years)**		
0–4	154	74.76
5–9	25	12.14
≥10	27	13.11

**Figure 1:**
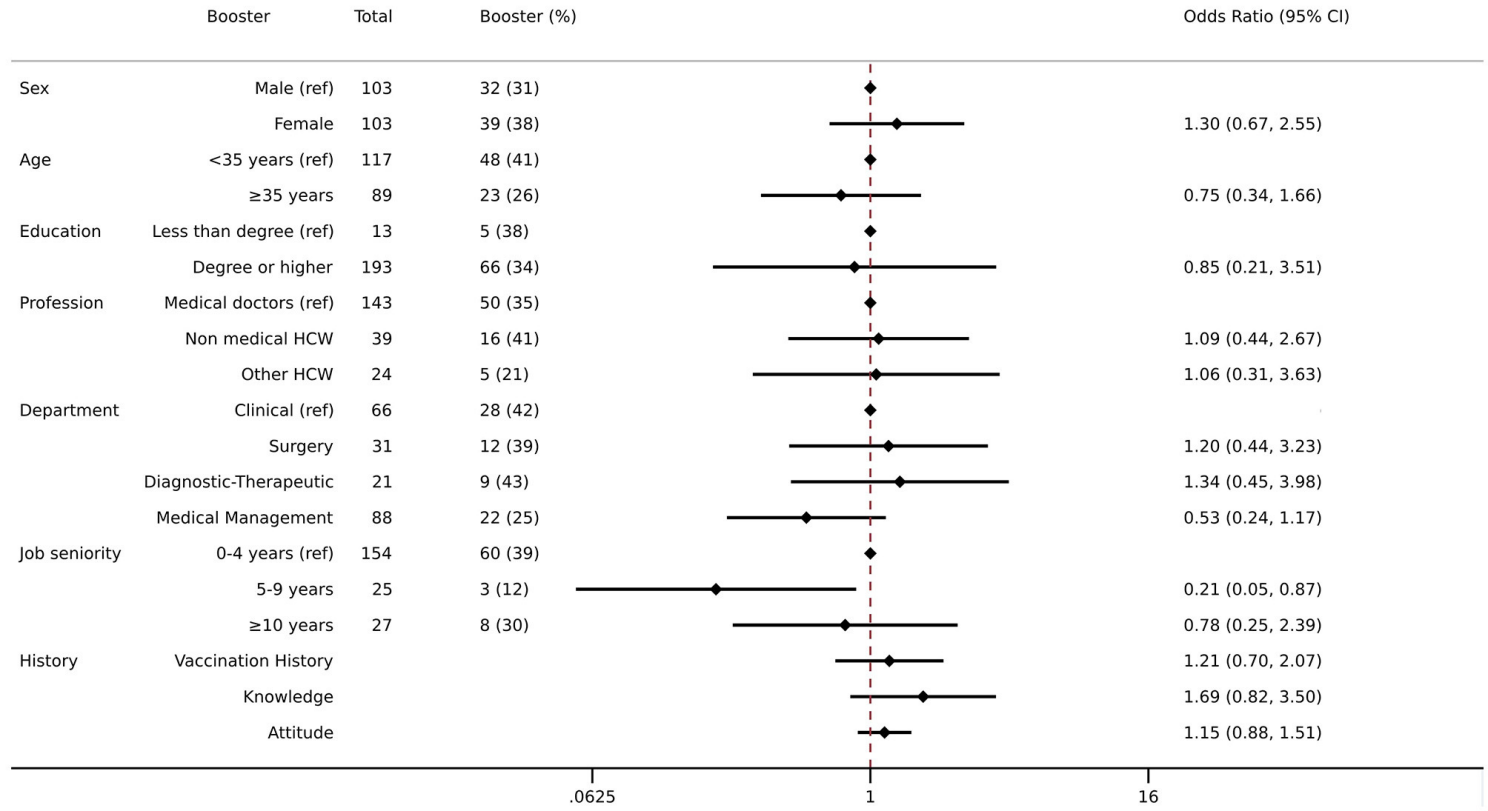
Association between demographic, job status, knowledge, attitude, vaccination history, and booster dose for Tdap. Multivariate logistic regression was employed including Tdap booster as an outcome variable and controlled for the following variables: sex, age, education, profession, department, job seniority, vaccination history, knowledge, and attitude. The results are presented as odds ratios (ORs) and 95% confidence intervals (95%CIs). The left column shows the crude number of those who received the booster Tdap and the proportion of them among the total.

One-third of the sample (34.5%) had a Tdap vaccination booster over the past 10 years. The results from the multivariable logistic regression model showed that as compared with those with 0–4 years of employment at a university hospital, those with 5–9 years of job seniority had a 78% lower likelihood of being vaccinated with the booster dose (5–9 years—OR: 0.22, CI: 0.06 | 0.85) (Figure 1).

**Figure 2:**
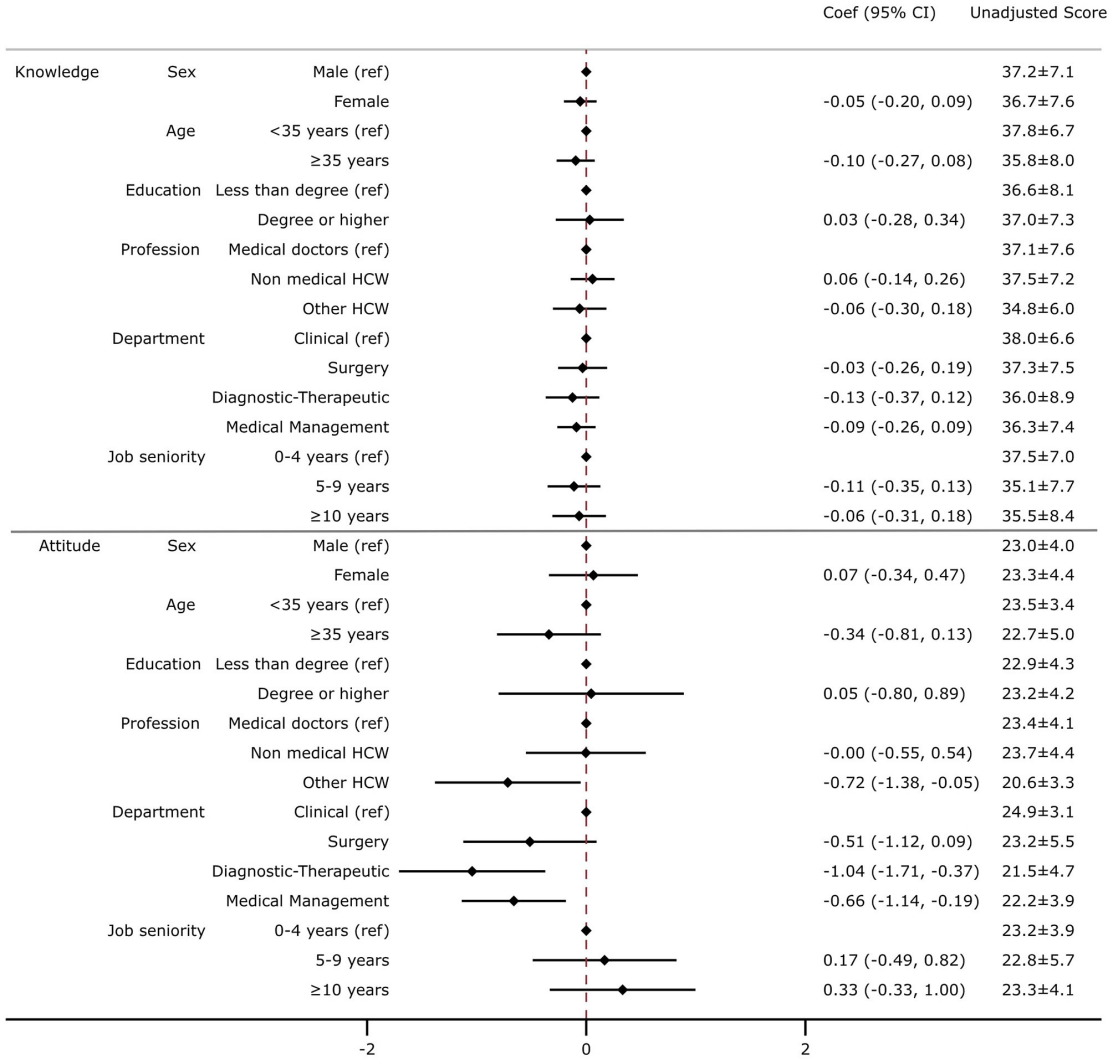
Association between demographic, job status, and knowledge or attitude toward vaccination. Multivariate linear regression was employed including knowledge **(top)** or attitude **(bottom)** as an outcome variable and controlled for the following variables: sex, age, education, profession, department, and job seniority. The results are presented as a coefficient (Coef) and 95% confidence intervals (95% CIs). In the right column, the unadjusted average score and standard deviation for attitude and knowledge are displayed. The knowledge score was calculated considering the average score of 15 questions regarding recommended vaccination (with a score ranging from 1 to 3 for each question, with a final score ranging from 15 to 45). The attitude score was calculated considering the average score of three questions regarding the perception of the risk of contracting an infection and the usefulness of vaccination for HcWs to protect themselves and patients (with a score ranging from 1 to 10 for each question, with a final score ranging from 3 to 30).

The average knowledge score was 36.94 (CI: 35.93|37.95) out of 45. No differences in the average knowledge score were found between sub-groups (Figure 2). The average attitude score toward vaccination was 23.16 (CI: 22.59| 23.73) out of 30. When compared with medical doctors, other HcWs had a lower attitude score of 2.2, on average, (other—Coef. −2.15 on 30; CI: −4.14 | −0.15) and when compared with those who worked in a clinical department, on average, those who worked in a diagnostic–therapeutic department or medical management had lower attitude scores of 3.1 and 2.0, respectively (diagnostic–therapeutic—Coef. −3.12 on 30, CI: −5.13 | −1.12; medical management—Coef. −1.98 on 30, CI: −3.41 | −0.56) (Figure 2).

## Discussions

In our cross-sectional study, conducted in the University Hospital “Federico II” of Naples, the largest university hospital in Southern Italy, we found that only one-third (34.5%) of the study population had a booster vaccination for Tdap, with a lower likelihood of receiving a booster dose for those with a 5–9 year employment history when compared with those employed for <5 years. No differences were found regarding the vaccination knowledge between sub-groups, while attitude toward vaccination was lower in the other HcWs (administrative employees, biologists) when compared with medical doctors and in HcWs employed in diagnostic–therapeutic and medical management departments when compared with clinical departments.

Overall, the prevalence rate of Tdap booster vaccination in the sample was as low as 34.47%. This evidence, although in the lower range, has been reported in similar studies conducted in the USA with values ranging from 34.7 to 47.2% (27–29) and in Turkey (36% of HcWs with at least one booster dose in the past) (30). Interestingly, we found no sex differences in the proportion of Tdap boosters received, although the previous literature suggested that HcWs of the female sex were more likely to receive the Tdap (31–33). We also found a weak positive association between younger age and the likelihood of Tdap booster vaccination (Supplementary Table 1), which, however, was not confirmed in the fully adjusted model. However, this evidence has been confirmed by previous studies conducted in similar settings (24, 29, 34, 35).

In the partially adjusted model, younger participants, as compared with those participants of 35 years and older, had a higher knowledge regarding recommended vaccines for the HcWs (Supplementary Table 2), although this was a weak association not confirmed in the fully adjusted model. This finding might be explained by the shorter time period since obtaining their degree. Furthermore, this evidence is consistent with a study conducted in similar settings (36).

Attitude toward vaccination varied according to occupation. In line with previous evidence (29), we found that medical doctors had significantly higher attitude than other HcWs, which might also be explained by their perception of being at high risk and the frequency of contacts with other high-risk groups, i.e., patients (37). We also found that attitude toward vaccination was higher for HcWs working in clinical departments, where the intensity of contact with high-risk patients is higher when compared with those working in diagnostic and medical management departments, which is in line with recent evidence conducted in similar settings (30, 38–41).

We conducted our research on HcWs working in the largest university hospital in Southern Italy. Hence, the results might be generalized to similar healthcare settings in the country. However, several considerations merit discussion. First, responses may be influenced by difficulty in recalling their vaccination status, particularly for pediatric vaccinations. However, when recall bias is equally distributed in every study participant, the overall effect of the bias on study findings is reduced (42). Second, although the questionnaire was designed to be anonymous, responses or the lack of participation may have been influenced by the fear of the vaccinations or being targeted for vaccination campaigns, especially after the COVID-19 pandemic and the decision by the Italian NHS to enforce the COVID-19 vaccination for HcWs. Third, this specific analysis was based on a relatively small sample, and the results might be influenced by possible selection bias, as only personnel more willing to share their experiences might have decided to participate. Finally, another limitation of the study was to assess knowledge in a yes-no-don't know system. Although this approach might limit the precision of the outcome derivation, this choice was made to avoid altering the original questionnaire.

## Policy

Healthcare workers are a high-risk population for infectious disease exposure and transmission. Low vaccine coverage for HcWs can lead to severe disease outbreaks, decreasing productivity, increasing absenteeism, and is also costly to the health system (43–46). Improving attitude and belief regarding vaccination among HcWs is important to avoid drops in the vaccination coverage rates and may also influence patients' responses to immunization campaigns (47). Our findings highlighted the importance to implement effective information and communication strategies, mostly among more experienced staff, to refresh and update information regarding vaccination in HcWs. Specifically, tailored strategies should be undertaken to improve Tdap booster coverage because, although the booster is offered free of charge in line with the national vaccination plan, there is no monitoring strategy in place as the quantitative serum immunoglobulin test is not included as a minimum requirement in the protocol of health surveillance for HcWs.

## Conclusion

In the present study, we found that only one-third of the HcWs employed at the University Hospital “Federico II” of Naples, the largest academic hospital in Southern Italy, had a Tdap vaccination booster in the past 10 years. Longer employment history was associated with a lower likelihood of receiving the Tdap booster. Medical doctors had a higher attitude toward vaccination than other HcWs. Our findings support the need to implement public health strategies to improve information and awareness toward vaccinations and specifically highlight the importance of actively including the Tdap booster every 10 years as a prevention tool to protect high-risk populations.

## Data availability statement

The raw data supporting the conclusions of this article will be made available by the authors upon request and approbation from the ethical committee.

## Ethics statement

The studies involving human participants were reviewed and approved by University Hospital Ethical Committee of “Federico II”. The patients/participants provided their written informed consent to participate in this study.

## Author contributions

RP and MT conceived the study and devised the study methodology and supervised the study. MM, CF, FE, MS, PM, and AP contributed to the acquisition of data for the study. RP and MM performed the formal data analysis. RP, MM, CF, FE, and MS wrote the first draft of the manuscript. RP had final responsibility for the decision to submit for publication. All authors reviewed and edited the manuscript, contributed to the article, and approved the submitted version.
