# Supplementary figures and images for: Knowledge and attitude factors associated with the prevalence of Tdap (tetanus, diphtheria, and acellular pertussis) booster vaccination in healthcare workers in a large academic hospital in Southern Italy in 2022: a cross-sectional study
Source: Front Public Health. 2023 Jul 13;11:1173482. doi: 10.3389/fpubh.2023.1173482 (PMC10374026; doi:10.3389/fpubh.2023.1173482)

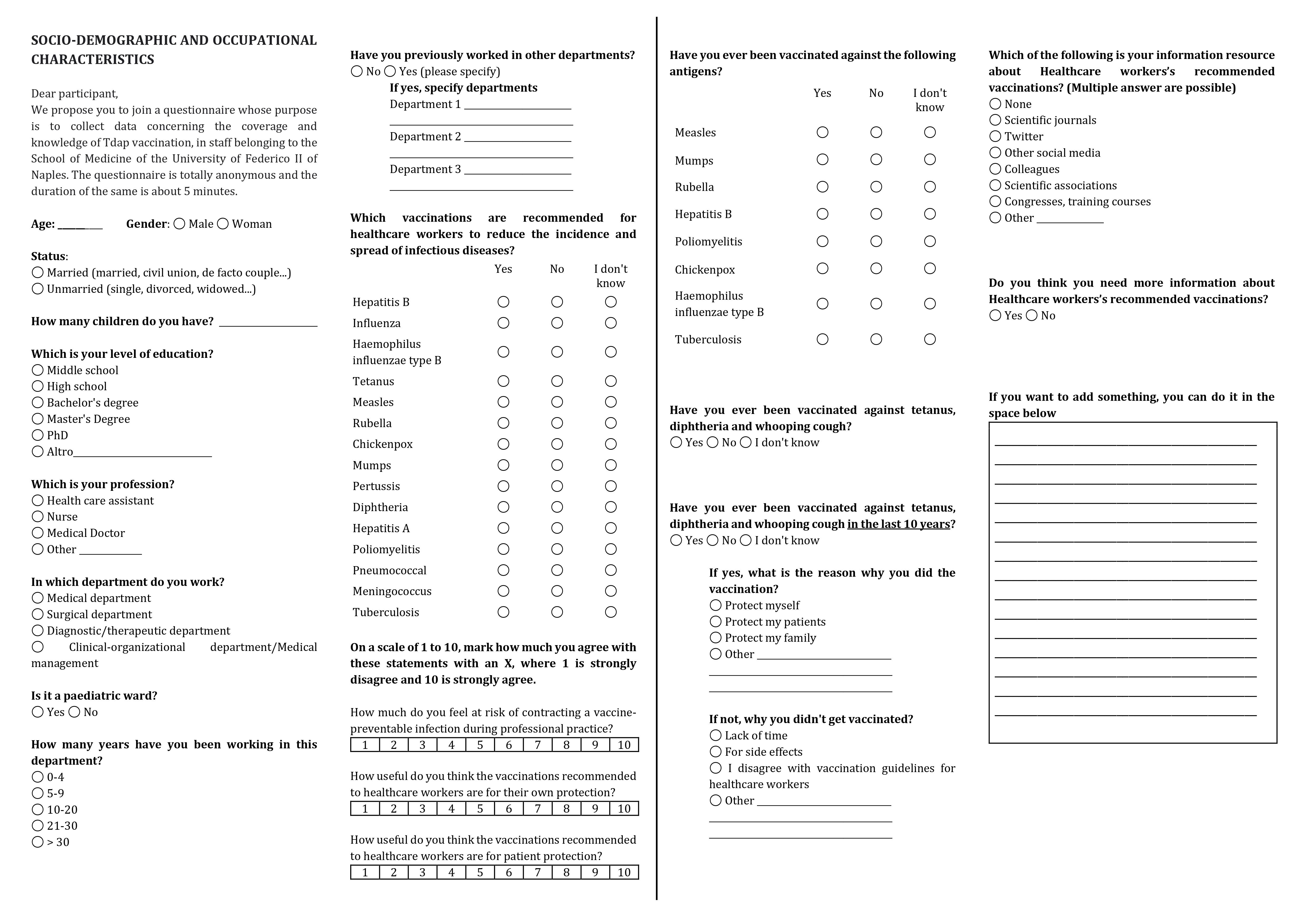

Supplement: Supplementary file 1 [file Image_1.JPEG]
