# Supplementary material for: Knowledge and attitude factors associated with the prevalence of Tdap (tetanus, diphtheria, and acellular pertussis) booster vaccination in healthcare workers in a large academic hospital in Southern Italy in 2022: a cross-sectional study
Source: Front Public Health. 2023 Jul 13;11:1173482. doi: 10.3389/fpubh.2023.1173482 (PMC10374026; doi:10.3389/fpubh.2023.1173482)
Supplement: Supplementary file 2 [file Data_Sheet_1.docx]

Supplementary Material

Knowledge and attitude factors associated with prevalence of Tdap (Tetanus, diphtheria, acellular pertussis) booster vaccination in healthcare workers in a large academic hospital in Southern Italy in 2022: a cross-sectional study

Michelangelo Mercogliano, Claudio Fiorilla, Federica Esposito, Michele Sorrentino, Pasquale Domenico Mirizzi, Antonio Parisi, Andrea Tajani, Gaetano Buonocore, Maria Triassi, Raffaele Palladino*

*** Correspondence:** Raffaele Palladino: raffaele.palladino@unina.it

# Supplementary Figures and Tables

## Supplementary Tables

| ***Booster*** | ***Partially Adjusted I*** | | | ***Partially Adjusted II*** | | |
| --- | --- | --- | --- | --- | --- | --- |
|  | ***OR*** | ***95%*** | ***C.I.*** | ***OR*** | ***95%*** | ***C.I.*** |
| ***Anagraphical Datas*** |  |  |  |  |  |  |
| ***Sex*** |  |  |  |  |  |  |
| *Male (ref)* |  |  |  |  |  |  |
| *Female* | *1.19* | *0.66* | *2.16* | *1.30* | *0.67* | *2.53* |
| ***Age*** |  |  |  |  |  |  |
| *<35 years old (ref)* |  |  |  |  |  |  |
| *≥35 years old* | *0.52* | *0.28* | *0.96* | *0.73* | *0.33* | *1.58* |
| ***Education and Work*** |  |  |  |  |  |  |
| ***Education*** |  |  |  |  |  |  |
| *Less than degree (ref)* |  |  |  |  |  |  |
| *Degree or higher* |  |  |  | *0.85* | *0.21* | *3.43* |
| ***Profession*** |  |  |  |  |  |  |
| *Medical doctors (ref)* |  |  |  |  |  |  |
| *Non medical HcW* |  |  |  | *1.14* | *0.47* | *2.73* |
| *Other HcW* |  |  |  | *0.81* | *0.25* | *2.68* |
| ***Department*** |  |  |  |  |  |  |
| *Clinical (ref)* |  |  |  |  |  |  |
| *Surgery* |  |  |  | *1.17* | *0.45* | *3.08* |
| *Diagnostic-therapeutic* |  |  |  | *1.05* | *0.37* | *2.96* |
| *Medical Management* |  |  |  | *0.48* | *0.22* | *1.04* |
| ***Job seniority:*** |  |  |  |  |  |  |
| *0-4 years (ref)* |  |  |  |  |  |  |
| *5-9 years* |  |  |  | *0.22* | *0.06* | *0.85* |
| *10+ years* |  |  |  | *0.77* | *0.26* | *2.32* |

**Supplementary Table 1.** Association between demographic, job status, and booster dose for Tdap. Multivariate logistic regression was employed including Tdap booster as outcome variable and controlled for the following variables: sex and age in model partially adjuster I and partially adjuster II and education, profession, department, and job seniority in model 2. Results are presented as odds ratios (OR) and 95% confidence intervals (95% CI).

| ***Partially Adjusted Model*** | ***Knowledge*** | | | | ***Attitude*** | | | |
| --- | --- | --- | --- | --- | --- | --- | --- | --- |
|  | ***Coef*** | ***95%*** | | ***C.I.*** | ***Coef*** | ***95%*** | | ***C.I.*** |
| ***Anagraphical Datas*** |  |  |  | |  |  |  | |
| ***Sex*** |  |  |  | |  |  |  | |
| *Male (ref)* |  |  |  | |  |  |  | |
| *Female* | *-0.06* | *-0.20* | *0.08* | | *0.06* | *-0.33* | *0.45* | |
| ***Age*** |  |  |  | |  |  |  | |
| *<35 years old (ref)* |  |  |  | |  |  |  | |
| *≥35 years old* | *-0.15* | *-0.28* | *-0.01* | | *-0.28* | *-0.68* | *0.11* | |

**Supplementary Table 2.** Association between demographic, and knowledge or attitude towards vaccination. Multivariate linear regression was employed including knowledge (left) or attitude (right) as outcome variable and controlled for the following variables: sex, and age. Results are presented as coefficient (Coef) and 95% confidence intervals (95% CI).

## Supplementary Figures

**Supplementary Figure 1.** Questionnaire administered for the present study. (A) front page, (B) back page.
